# Variants of active assist robotic therapy: Feasibility of Virtual Assistance and Proprioceptive Training as gauged by their effects on success and motivation during finger movement training after stroke

**DOI:** 10.21203/rs.3.rs-5702495/v1

**Published:** 2025-04-15

**Authors:** Andria Farrens, Dylan Reinsdorf, Luis Garcia-Fernandez, Raymond Rojas, Vicky Chan, Joel Perry, Eric Wolbrecht, David Reinkensmeyer

**Affiliations:** University of California, Irvine; University of California, Irvine; University of California, Irvine; University of California, Irvine; University of California, Irvine; University of Idaho; University of Idaho; University of California, Irvine

## Abstract

**Background:**

Standard robotic therapy for upper extremity stroke rehabilitation provides physical assistance during video game-based training. While effective, it requires complex equipment, focuses on visuo-motor control, and yields variable results. This raises the question whether more pragmatic or targeted variants can be developed. To explore this, we pilot tested two novel robotic therapy modes. In the Virtual Assistance mode, game parameters are modulated to promote task completion without physical assistance – potentially useful when only sensors are available, although its acceptability for people with more severe motor impairment is unclear. In the Proprioceptive Training mode, participants play video games through a combination of visual and haptic cues. This approach aims to retrain proprioception, but may also be too challenging for individuals with proprioceptive impairment to find it motivating. This study tested the feasibility of both variants across a range of motor and proprioceptive impairments, comparing them to training with Standard robotic therapy.

**Methods:**

Chronic stroke participants (N = 46) were randomized to receive Standard, Virtual, or Proprioceptive Training. Participants used the FINGER robot to train in three sessions across one week, during which an adaptive algorithm attempted to titrate success to ~ 80%. Baseline proprioceptive and motor function were assessed prior to training, and motivation for training was assessed using the Intrinsic Motivation Inventory. Feasibility was evaluated by levels of gameplay success and motivation for training.

**Results:**

Participants of widely varying motor and proprioceptive ability achieved ~ 80% success for all three modes. However, Virtual Assistance resulted in significantly diminished motivation, due to lower perceived competence when participants were not provided with physical assistance. Participants with impaired proprioception rated Proprioceptive Training engaging, although it was more challenging for them and they required an increased level of assistance.

**Conclusion:**

Both training paradigms were feasible for use with chronic stroke survivors and were able to achieve high gameplay success and motivation for training. However, physical assistance appeared to have an advantage over Virtual Assistance in raising training motivation. Proprioceptive Training required high levels of assistance, but was motivating even for people with poor proprioception.

## Introduction

The first robotic therapy device, MIT-MANUS, focused on providing physical assistance to the arm as the patient played a simple video game comprised of reaching to targets [1], [2]. Since then, various related paradigms have emerged, incorporating alternate forms of active assistance—such as gravity counterbalancing [3]-[6] and adaptive controllers [7]-[12]—and a wide variety of gaming interfaces, including games that encourage movement exploration [13], [14], utilize error augmentation [15]-[18], or incorporate haptic simulation [19]-[22]. However, the basic paradigm of active assistance during visually-driven, video game play (which we will call “Standard Robotic Training”) has been the most frequently implemented, tested, and commercialized paradigm for robotic therapy.

In Standard Robotic Training, the robotic device detects the patient's movement intent and provides targeted support as needed to help them complete the motion, ensuring active participation across a range of impairment levels and providing an experience of movement success. Systematic reviews indicate that training with this paradigm after stroke modestly reduces upper extremity (UE) impairment, with benefits comparable to or slightly exceeding a matched dose of conventional movement therapy [23]-[27]. However, outcomes vary substantially between patients, with one possible reason being that the ability to benefit depends on the availability of accurate proprioceptive input [28],[29]. Further, it is unclear whether the robotic actuators are needed for the therapeutic benefit, as training with sensor-based systems can also facilitate active participation, provide reward, and produce clinical benefits [30], [31].

Thus, an important question is whether variants of robotic therapy can be developed that are more pragmatic (such as not requiring actuators) and/or better targeted to specific impairments (such as the proprioceptive impairments that may limit the effectiveness of robotic training). Here we report the development and feasibility testing of two robotic therapy variants aimed at reducing the complexity of robotic therapy and encouraging proprioceptive learning, respectively.

The first mode we tested, Virtual Assistance Training, removes reliance on physical assistance by instead adaptively modifying game parameters and adjusting the virtual representation of the participant’s movements to control success and provide positive reinforcement for movement attempts. Virtual Assistance Training would theoretically be useful when only a sensor is available, but it is unclear how individuals with more severe motor impairment will engage with training in the absence of physical assistance. We found previously that lowering amounts of robotic assistance during robotic therapy reduced self-reported levels of motivation, however lower assistance was also tied to lower gameplay success, making the relative contribution of success and assistance to motivation unclear [28].

The other robotic therapy variant we tested here, Proprioceptive Training, replaces visual elements of the video games with proprioceptive cues, a strategy we call “propriopixels” ([32]), that is aimed at retraining proprioceptive awareness. Over half of individuals in the chronic phase of stroke recovery exhibit proprioceptive deficits in the hand [33]. We recently found that Standard Robotic Training of the hand was less effective for individuals with baseline finger proprioceptive deficits [28]. A wide variety of studies have demonstrated that proprioceptive learning is possible and exhibits several features similar to motor learning [34], [35]. Targeting proprioception during robotic therapy might be useful for retraining proprioception, but it is unclear whether individuals with severe proprioceptive impairment will find it motivating, since they are being asked to make gameplay decisions based on a sensory input that they may struggle to perceive.

Here, we present the results of an experimental study aimed at evaluating the feasibility of training with these novel strategies compared to Standard Robotic Training. Forty-six people in the chronic phase of stroke recovery (> 6 months post-stroke) were randomized to train with one of the techniques three times in one week. An adaptive algorithm was used to try to titrate success to ~ 80% for each training mode. We hypothesized that the algorithm would be able to adapt levels of success for individuals with a wide range of motor and proprioceptive impairments in all paradigms. Further, we hypothesized that the high success levels endowed by the assistance would lead individuals to rate the training modes as motivating, providing support for their feasibility for long-term training.

## Methods

### Participants

Participants were recruited from the UCI stroke survivor database, regional hospitals and stroke support groups. Inclusion criteria were: aged 18–85 years old, with stroke (single or multiple, ischemic or hemorrhagic) confirmed via radiological imaging occurring greater than 6 months prior to enrollment with residual unilateral upper extremity weakness. Participants were excluded if they scored fewer than 3 blocks on the box and blocks test (BBT) or had less than a 20% difference in BBT score between their affected and unaffected arm. Additional exclusion criteria included severe muscle tone of the affected arm (score > 3 on the Modified Ashworth Spasticity Scale), severe aphasia (score of 3 on question 9 of the NIH Stroke Scale), evidence of major depression (DSM V criteria or Geriatric Depression Scale score > 10) and concurrent participation in another study related to stroke recovery. A total of 46 chronic post-stroke adults were enrolled in this study. All visits took place at UCI. All participants provided informed consent (UCI IRB#476), and procedures were conducted according to the Declaration of Helsinki. The trial was registered on ClinicalTrials.Gov (NCT04818073).

### Experimental protocol

**Figure 1:**
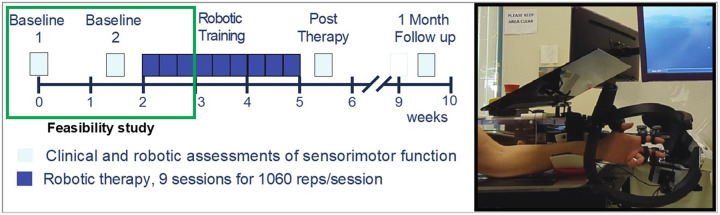
Left: Experimental protocol of the parent randomized, controlled trial. Data from the baseline sessions and first week of training (green box) were used in this study to evaluate feasibility of each training mode, as judged by success and motivation. The baseline sessions included clinical assessments of hand function (Box and Blocks, Fugl-Meyer) and robotic assessments of sensorimotor function ( finger capacity, finger and thumb proprioception). Each robotic training session included 10 games of RehabHero (5 in two-string, 5 in three-string mode), and 18 games of FingerPong (8 in matched mode, 10 in target mode). Physical assistance was tuned the first session, and unassisted gameplay was assessed the third session. Right: The FINGER robot can support flexion and extension of the index and middle finger, and adduction/abduction and flexion/extension of the thumb, shown here attached to a participant’s left hand. The black plastic occlusion screen slides over to cover the participants view of the hand during training and assessments. FINGER can be used to train either hand in mirrored right and left robotic configurations.

In this paper, we report on the game-play success and motivation data collected from the first week of an ongoing clinical trial of robotic hand therapy for chronic stroke survivors (Fig. 1). Full clinical outcomes will be published in a subsequent paper. All participants trained using the FINGER robotic exoskeleton, which can assist and measure flexion and extension of the index and middle finger, and adduction/abduction and flexion/extension of the thumb [36], [37]. “Baseline” clinical and robotic evaluations of hand sensorimotor function were performed twice, one week apart, prior to starting robotic training. At the second Baseline visit, participants were randomly assigned to one of three training groups; Standard Training, Proprioceptive Training, or Virtual Training (detailed below), and were briefly trained on FINGER games to familiarize them with task instructions. The following week, participants then trained for three, 2-hour sessions with FINGER. For all groups, each training session consisted of 10 games of RehabHero (5 in two-string mode, 5 in three-string mode, described next), and 18 games of FingerPong (8 in classic mode, 10 in target mode, detailed below), resulting in 1060 cued flexion/extension movements in the session.

### Rehabilitation games

**Figure 2:**
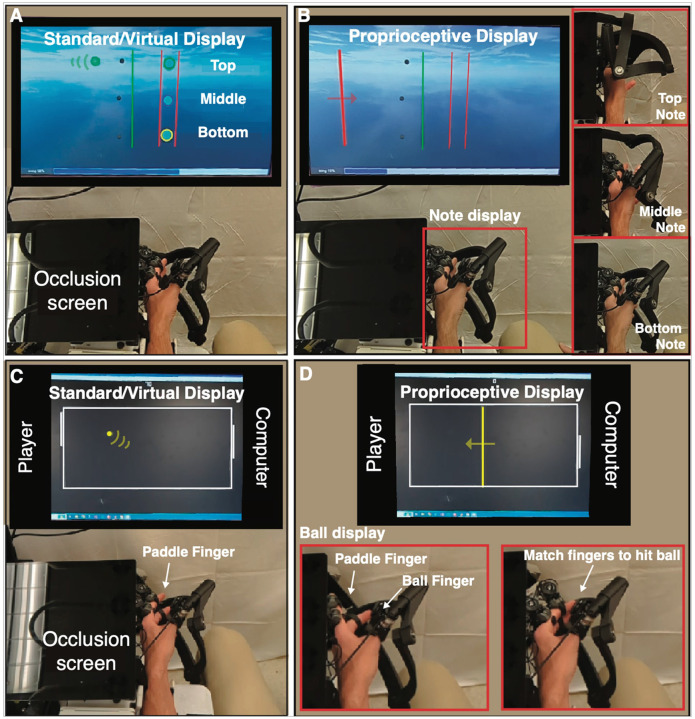
Overview of visual and proprioceptive games. In all training modes, vision of the hand is blocked from view by the black plastic occlusion screen. A: Visually guided RehabHero, in which the note (green ball) moves across the screen to the “fretboard” (3 vertical keys representing guitar strings) to indicate which finger (top key = index finger, middle key = both fingers, bottom key = middle finger) to flex to try to hit the note when it reaches the fretboard. Participants’ finger position is represented by the black dots. To train finger extension, the participants were cued to extend their fingers past the green vertical line after each note. B: Proprioceptive RehabHero, in which vision of the fretboard and incoming notes is removed. The incoming note’s vertical position is displayed proprioceptively by the position of the thumb, which is moved upward into radial abduction to indicate top key notes, a neutral middle position to indicate middle key notes, and downward into palmar abduction to indicate bottom key notes. The timing of the note arriving at the fretboard is displayed by a red line moving horizontally across the gaming monitor. C: Visually guided FingerPong, in which the player’s paddle and the ball are always visible. The player controls their paddle by flexing/extending their finger to move the paddle down/upward to hit the ball when it arrives on the player’s side of the gaming monitor. D: Proprioceptive FingerPong. The vertical position of the ball is removed and displayed proprioceptively by the robot moving the “ball finger” in flexion/extension to display the ball’s downward/upward position on the screen (left insert). The ball’s horizontal movement is displayed visually by a vertical yellow bar moving laterally across the screen. The participant must match their “paddle finger” to their “ball finger” when the yellow line reaches the player side of the monitor to hit the ball (right insert).

Participants in the Standard and Virtual Training groups played rehabilitation games in the Vision-based game mode, while participants in the Proprioception training group played in the Proprioceptive game mode (Fig. 2), detailed below. For all groups, the workspace for each game was bounded between 90% of the participant’s maximal voluntary flexion/extension in the device, with a minimum workspace of 30 degrees. In all groups, vision of the hand was occluded by a black plastic divider during training.

#### Vision-based games:

In RehabHero, a version of the popular musical video game GuitarHero (Fig. 2A), participants were cued to hit notes on one of three different “strings” by flexing their index and/or middle finger to stop inside of the target “string” as a scrolling note passed through it [36], [38]. Participants needed to flex their index finger to hit notes incoming on the top string, their middle finger to hit notes on the bottom string, and both fingers to hit notes on the middle string. In the protocol, the first five RehabHero songs used only top and bottom strings (2-string mode), while the second five used all three strings (3-string mode), increasing the challenge. In FingerPong, participants flexed or extended their finger to move a paddle up or down to hit a ball to a computer opponent in a modified version of the classic Pong game (Fig. 2C). In classic mode, participants could hit the ball with any part of their paddle; in target mode, participants were cued to hit the ball with a specific part of the paddle to rebound the ball to highlighted targets, increasing the challenge. Participants played half of the FingerPong games with their index finger controlling the paddle, and half with their middle finger.

#### Proprioceptive Gaming:

The Proprioceptive Training mode for both RehabHero and FingerPong was implemented by removing some of the visual information displayed on the screen and replacing it with physical cues, a robotic gaming strategy we call “propriopixels” [32]. In RehabHero, the incoming note was replaced by a vertical bar moving across the screen to indicate the timing the note arrives at the string, while occluding the vertical position of the note. We then displayed which string the incoming note was arriving on proprioceptively with movement of the thumb (Fig. 2B). The robot moved the thumb to a top position (corresponding to radial abduction) for top string notes, a bottom position (palmar abduction) for bottom string notes, and halfway between to indicate middle string notes. Because the hand was covered by an opaque screen during gameplay, participants had to sense their thumb position proprioceptively and use this information to decide which finger(s) to try to flex to hit the incoming note.

For FingerPong, we replaced the display of the ball’s vertical position on the screen with robot guided flexion/extension movement of one finger to display the ball’s (bottom/top) vertical position on the screen (Fig. 2D). Again, the ball’s horizontal position (hit timing) was displayed by a vertical bar moving across the screen. Participants were tasked with relaxing their “ball finger” and proprioceptively sensing its position and then moving the “paddle finger” (the finger controlling the paddle) to match its position. In the target mode, which we designed to be more challenging, participants had to move the paddle finger into relative positions to the ball finger (slightly above, centered, slightly below) to hit the ball to targets on the other side of the screen.

#### Robot Assistance Strategy:

In the previous FINGER clinical trial [28], we found that an 80% success rate yielded higher self-reported motivation than a 50% success rate. Similarly, our study of a home-based sensor system for unassisted movement training indicated that a game success rate of 80–90% was predictive of the best perseverance, with higher and lower success rates showing lower perseverance [30]. Thus, we chose to adjust the robot and game parameters to try to achieve 80% game success; these parameters depended on the game and assistance mode, as described below.

To achieve parameters that stabilized game success at 80%, the assistive algorithm (Eq. 1) increased the parameters by a relative increment of 1 for each missed movement (i.e., note missed) and decreased by ¼ for each successful movement (i.e., ball hit) as done in our previous study [39]. Using this algorithm, when the participant reaches 80% success, the tuning gain converges to a stable value.

(eq. 1) Gain(n+1)={Gain(n)+1xifmissedGain(n)−14xifhit}

where n is the current trial number (eg. note in guitar hero), and x is an empirically determined incremental value to adjust parameter gains.

Physical assistance was implemented by the FINGER robot in the same way as the original study. Briefly, the FINGER robot applied assistive forces using a compliant position controller with a tunable gain to guide the fingers along a smooth trajectory to intercept the note at the cued time, or to move the paddle to intercept the ball. Assistance was only provided if the participant initiated movement themselves, as determined via force sensors mounted at the connection point between the fingers and the FINGER exoskeleton (threshold = 2N). Gains were increased for each missed note/ finger extension in RehabHero or missed hit in FingerPong, and decremented for successful movements. In RehabHero, assistance gains were finger and direction specific; in FingerPong, assistance was bidirectional and averaged across fingers. This physical assistance strategy was used for the “Standard Training” group and the “Proprioceptive Training” mode.

In this work, we extended this algorithm to adjust game parameters rather than physical assistance gains to create the “Virtual Training” mode. In this group, we provided virtual assistance during visually guided gaming, by amplifying the virtual representation of participants’ movements on the screen and by adjusting the required timing accuracy. In RehabHero, movement amplification was achieved by setting an inflection point halfway between participants’ maximum flexion and extension and applying a tunable linear gain such that the representation of flexion and extension movements made on either side of the inflection point were amplified. Timing constraints were adjusted independently from movement gains; missed notes resulted in both movement and time adjustments, while instances in which the participant hit the note outside of the correct time window resulted in adjustments to the timing constraint alone. In FingerPong, Virtual Assistance was achieved by adjusting the paddle size and speed of the ball, such that missed hits increased the paddle size and reduced the ball speed, while successful hits resulted in decreased paddle size and increased ball speed. Other than the graphical scaling of finger to game movement, the visual interface of the games in physical and virtual assistance modes were identical.

Across all gaming modes, we further adjusted our algorithm to allow for increased virtual challenge if the participant exceeded 80% success rates without any assistance. This was done as success rates > 90%, in which training is “too easy” have been shown to reduce motivation for training [31]. Virtual challenge was implemented by increasing the required timing accuracy in RehabHero, and by increasing the speed of the ball and decreasing the paddle size in FingerPong. The gains for each game were tuned in the first session of each week to prevent slacking and then held constant for the remainder of the week for training.

### Performance Metrics

#### Training Performance:

Performance during training was quantified by participants’ average success rate in each gaming mode across sessions 2 and 3 that were performed with fixed assistance gains. Unassisted gameplay was evaluated at the beginning of session 3, in the 2-string mode for RehabHero and the classic mode for FingerPong, to compare with assisted gameplay. We additionally quantified the magnitude of assistance needed in each gaming mode, as well as the number of movements attempted across the three training sessions.

#### Robotic Assessments of Motor and Proprioceptive Ability:

We assessed participants’ finger strength and proprioceptive ability using a battery of robotic assessments performed in the baseline visits. We collected these metrics to ultimately understand how baseline impairments relate to the therapeutic efficacy of training, which we will report in a companion paper. For this paper, we used them to understand how the adaptive algorithm was able to titrate success as a function of motor and proprioceptive impairment. All proprioceptive assessments were performed with vision of the hand occluded by a black plastic occlusion screen (Fig. 1).

To quantify the motor capacity of participants fingers, which we expected to impact feasibility of training for the Virtual Assistance Training group, we measured participants’ maximum voluntary contraction (MVC) in flexion and extension for both the index and middle fingers when moved independently and in tandem. We then quantified motor ability using a previously developed metric, “hand capacity” that creates a summary score that combines patients finger strength and ability to move the fingers independently [40]. Higher scores coincide with greater strength and individuation.

The primary proprioceptive assessment was Crisscross, which was developed for our previous clinical trial with FINGER, and was shown to be predictive of responsiveness to Standard Robotic Training [28], [33], [41]. In Crisscross, participants are tasked to push a button at the instance of perceived finger crossing as the robot moves their fingers back and forth in an alternating, crossing pattern. In this study, Crisscross had 20 total crossings, occurring at speeds ranging from 8–18 deg/s per crossing in a pseudorandom order. Performance was quantified by the average absolute error between fingers at the instant of button press.

The next two assessments, ThumbSense and Move and Match, were designed to be matched to the proprioceptive-elements of Proprioceptive Training, to assess participants proprioceptive ability independently from a gamified setting. ThumbSense, assessed participants’ ability to discern between the thumb positions used in the proprioceptive RehabHero mode (detailed above). The thumb was randomly moved to the top, middle, and bottom string positions 20 times in a pseudorandom order at the same speed as used in training. The robot paused for 6–10 seconds in each position, matched to the duration allowed for position discernment during gameplay. Participants were instructed to state their thumb position each time it came to rest and were scored based on their accuracy. The second assessment, Move and Match, tasked participants to move one finger (index/middle) to track robot-facilitated movements of the other finger (middle/index). Physical performance in this assessment mimicked the tracking and matching of the ball finger in the proprioceptive FingerPong game (detailed above), and commonly used joint-reproduction assessments [34], [42]-[44]. Performance was quantified as the average absolute tracking error between fingers. Note that Move and Match performance depended not only on proprioceptive ability, but also on finger movement ability. For a more direct measure of finger proprioceptive ability alone, we used Crisscross.

#### Clinical Assessments:

In addition to robotic assessments, we performed standard clinical tests of hand function at the baseline visits, including the Box and Block test, which scores the number of blocks transferred over a divider within one minute by the affected arm [45], and the upper extremity Fugl-Meyer (UEFM) assessment [46].

#### Motivation Assessment:

Finally, to evaluate participants motivation in each training mode (Standard, Virtual, Proprioceptive) we had them evaluate their motivation for training using an abbreviated version of the Intrinsic Motivation Inventory [47], identical to the version we used in the previous study of FINGER [28]. The survey included 14 questions evaluating participants perceived Value/Usefulness of the task, Pressure/Tension while performing the task, Effort/Importance of the task, Interest/Enjoyment of the task, and their perceived Competency (Supplemental Fig. 1). Participants evaluated their IMI at the end of the first training session, after completing 10 RehabHero games and 18 FingerPong games.

#### Data Analysis:

We used Kolmogorov-Smirnov testing to determine the normality of data. For normal distributions, we used two sample and paired t-tests for comparison testing, and for non-normal data, we used Wilcoxon rank and signed rank testing.

To determine the efficacy of the assistance algorithm in titrating success within each group, we quantified participants average success and level of assistance needed in each training mode across sessions 2 and 3, and compared participants average assisted performance to unassisted gameplay using comparison testing. Within each of the new training groups, we performed a correlational analysis to determine if the assistance strategy was able to mediate success in individuals with motor deficits ( finger capacity) in the Virtual Assistance group, and proprioceptive deficits (Move and Match, ThumbSense) in the proprioception group. Across all training groups, we further investigated how our primary metrics of sensorimotor hand function (Crisscross, Finger Capacity, BBT), that have previously been identified as predictive of training success [28], [40], related to game success in assisted and unassisted modes via correlation analyses.

Feasibility of each new training mode was compared to the Standard Training group. Specifically, we performed comparison testing between the new (Virtual, Proprioceptive) training groups to the Standard Training group, to identify any differences in game success achieved, the relative assistance needed in each mode, and their motivation. We further performed an exploratory correlation analysis to identify how game play success and sensorimotor hand function were related to motivation for training within and across all game modes.

## Results

Participants who were at least six months post-stroke (N = 46) with hand impairment were randomized to receive Standard Training (i.e., physical assistance with visual video games, N = 15), Virtual Training (i.e., assistance provided by adjusting game difficulty, N = 16), or Proprioceptive Training (i.e., physical assistance with propriopixels, N = 15) with the FINGER robot during three training sessions in which they played the games RehabHero and FingerPong. We used an adaptive algorithm to adjust training parameters to try to achieve a game success of 80% for all modes and games. Participants rated their motivation using the Intrinsic Motivation Inventory (IMI) following the initial training session. Participants additionally performed assessments of their finger and thumb proprioception, finger strength and individuation, the Box and Block Test (BBT), and the Upper Extremity Fugl-Meyer (UEFM) assessment. Table 1 provides an overview of these metrics for all participants. Standard deviations varied from 23%-100% of the arithmetic mean, indicating a wide range of sensorimotor ability in the participants sampled. There were no significant differences between training groups for any metric.

### Success modulation

**Figure 3:**
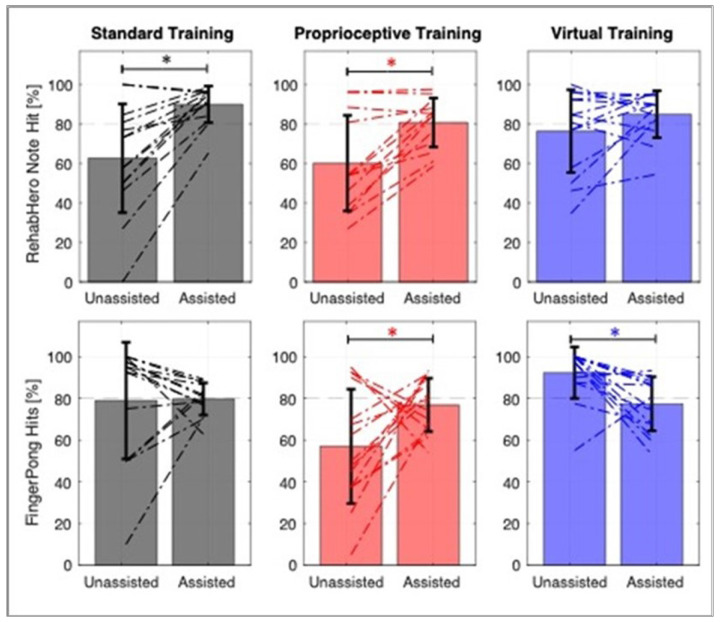
Success modulation for the three training groups in RehabHero (Top) and FingerPong (Bottom). Change in individual participant performance is indicated by the dashed lines. Error bars depict the mean and standard deviation across the group. Proprioceptive FingerPong had significantly lower unassisted success rates compared to the visually guided mode (Standard and Virtual; p < 0.01). Moreover, both visually guided modes had a greater proportion of participants exceeding the target 80% success rate, requiring virtual challenge to reduce their success in the assisted modes. Asterisks denote significant pairwise comparisons at p < 0.05.

Unassisted game success levels, assessed in the third training session, varied greatly across participants, ranging from ~ 10–100% (Fig. 3), consistent with participants’ wide range of sensorimotor ability (Tbl. 2). The adaptive assistance strategy we implemented to adjust the robot and game parameters succeeded in keeping gameplay performance within ~ 5% of 80% in all training modes and games, except for Standard Training in RehabHero, which approached 90% (Fig. 3; RehabHero success: Standard: 89.9 +/− 9.2; Proprioceptive: 80.7 +/− 12.4; Virtual: 84.9 +/− 11.9, Pong success: Standard: 79.7 +/− 7.7; Proprioceptive: 76.9 +/− 12.8; Virtual: 77.4 +/− 13.0). The assistance strategy was imperfect, as evidenced by the variability (standard deviations ~ 10%) in assisted success rates across participants.

For some participants, controlling success rates involved increasing assistance to increase success (Fig. 3). For others with high game success, controlling success rates meant making gameplay more difficult, particularly in the newly developed FingerPong game. Increasing game difficulty was made possible by use of the adaptive algorithm to increase the virtual gaming challenge.

Finally, proprioceptive gameplay modes had lower success levels than standard visually-guided gameplay. Specifically, playing unassisted FingerPong proprioceptively resulted in lower success than playing it visually (Wilcoxon T-test, p < 0.012), and proprioceptive RehabHero had significantly lower success than Standard Training when played with assistance (Wilcoxon T-test, p < 0.016), suggesting proprioceptive gaming was more challenging.

### Gameplay difficulty and magnitude of assistance

**Figure 4:**
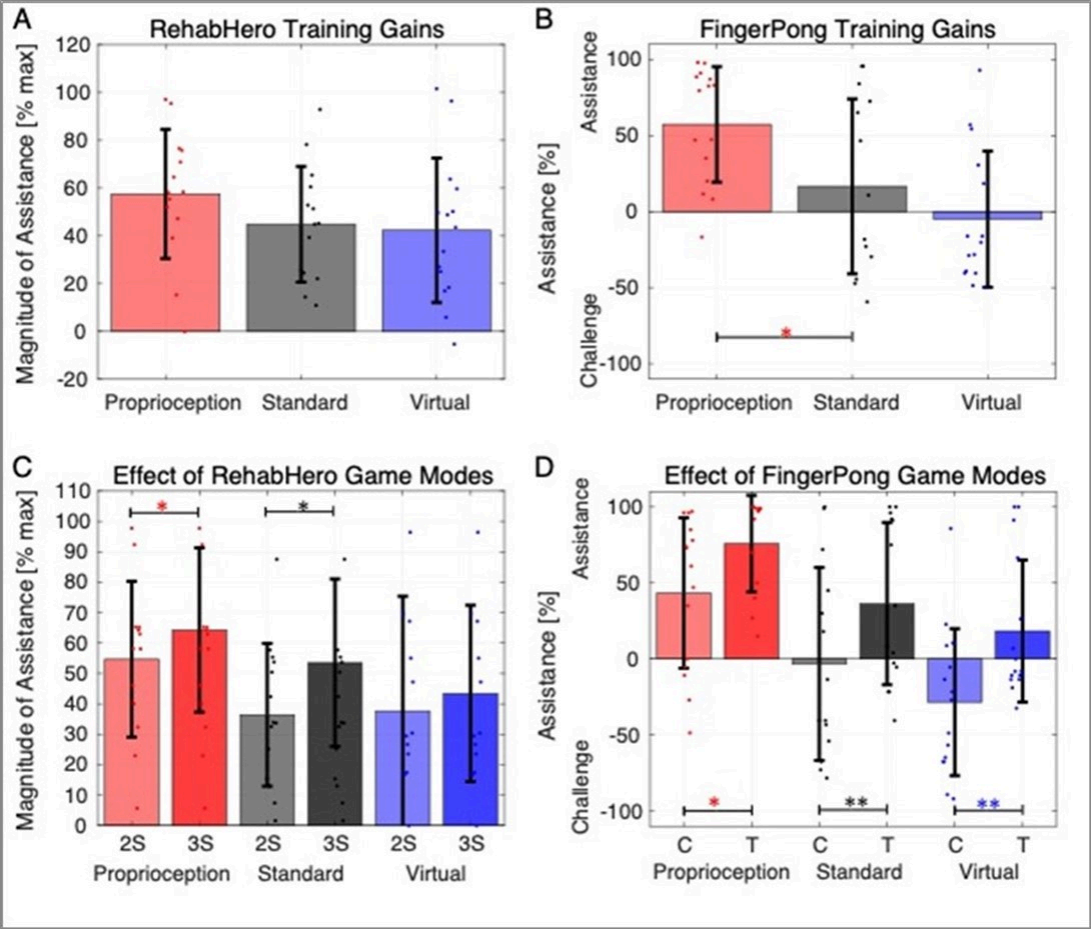
Assistance amounts depended on training and game modes. Top Row: The percent assistance required in all groups in RehabHero (A) and FingerPong (B), presented as a percentage of the maximum assistance possible. Bottom Row: Breakdown of assistance required for easy and hard game modes within each gaming paradigm. C: For RehabHero, 2-string mode is labeled as 2S, and 3-string mode as 3S. D: For FingerPong, classic mode is labeled as C, and target mode is labeled as T. Single asterisks denote significant Wilcoxon pairwise comparisons at p<0.05, while double asterisks denote p<0.001.

The Proprioceptive Training group needed relatively more assistance than the Standard Training group in both the RehabHero (Wilcoxon T-test, p < 0.051), and FingerPong games (Wilcoxon T-test, p < 0.032, Fig. 4B). In line with gameplay success results, this suggests that the proprioceptive gaming mode was more challenging compared to Standard Training.

Within each gaming mode, we found that the “harder” gaming modes (3-string for RehabHero, target mode for FingerPong) were indeed more challenging, evidenced by participants requiring significantly more assistance to achieve the desired 80% success rate (Fig. 4C,D). These modes represent another avenue for increasing gaming variety and challenge, that the assistance algorithm can modulate to maintain controlled, high levels of success.

### Assistance reduced the effect of sensorimotor impairments on gameplay success

Next, we investigated how baseline impairments in motor and proprioceptive ability impacted game play success with and without assistance, hypothesizing that the assistance strategies would compensate for participants’ impairment levels.

**Figure 5:**
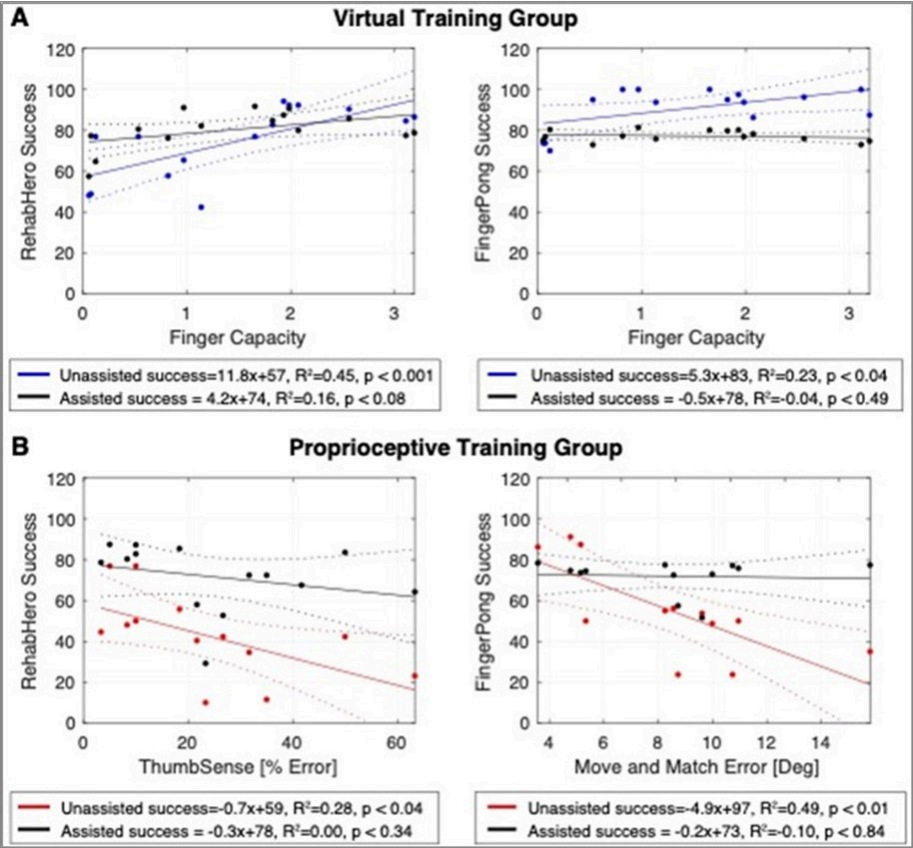
In the Virtual Training group (A), baseline finger capacity was significantly related to unassisted gameplay (shown in blue) success in both RehabHero and FingerPong. Virtual assistance, which modulates game parameters rather than providing physical assistance, was able to attenuate the dependence of gameplay success on baseline finger capacity, both in elevating success in RehabHero and attenuating success FingerPong (shown in black). In the Proprioceptive Training group (B), the proprioception assessments matched to the two games were significantly related to unassisted game success (shown in red), consistent with this mode targeting proprioceptive ability for gameplay. However, this dependence was attenuated by physical assistance (shown in black).

For the Virtual Assistance group, which provides no physical assistance and therefore may be unsuitable for individuals with severe motor impairment, we analyzed the relationship between finger capacity, a summary metric of participants finger strength and individuation, and unassisted and assisted gameplay success. We observed a significant dependence of unassisted success on finger capacity, but no significant relationship when assistance modulation was turned “on”, indicating that the virtual assistance algorithm was able to mediate success across a wide range of motor impairment levels (Fig. 5A). Interestingly, for FingerPong, the assistance strategy largely had to attenuate success levels for individuals with high finger capacity, suggesting that the initial settings for the classic FingerPong game may be too easy for most participants.

For the Proprioceptive Training group, we analyzed the relationship of the proprioceptive assessments most closely related with each game (i.e., ThumbSense assessment for RehabHero and Move and Match assessment for FingerPong) and gameplay success. As expected, finger and thumb proprioceptive ability predicted unassisted game success for the Proprioceptive Training mode, consistent with the reliance of these games on proprioception (Fig. 5B). However, with physical assistance, the significant correlation between baseline proprioceptive error and game success was eliminated. This indicates that even individuals with severe proprioceptive impairments were able to achieve high levels of success during Proprioceptive Training.

To further understand how hand sensorimotor function mediates success across all training modes, we repeated this type of analysis combining data for all three training modes and focusing on the relationship of game success to: 1) Crisscross error (our primary measure of finger proprioceptive ability), 2) Finger capacity (the measure of finger force production ability that we previously developed), and 3) BBT score (a measure of hand function). Crisscross error was independent from Finger Capacity (R^2^ = 0.075, p > 0.09), however, both were significantly correlated with BBT (Crisscross: R^2^ = 0.15, p < 0.009, Finger Capacity, R^2^ = 0.35, p < 0.001). We again hypothesized that the adaptive assistance provided for each mode would attenuate the effects of both proprioceptive and motor impairment.

**Figure 6:**
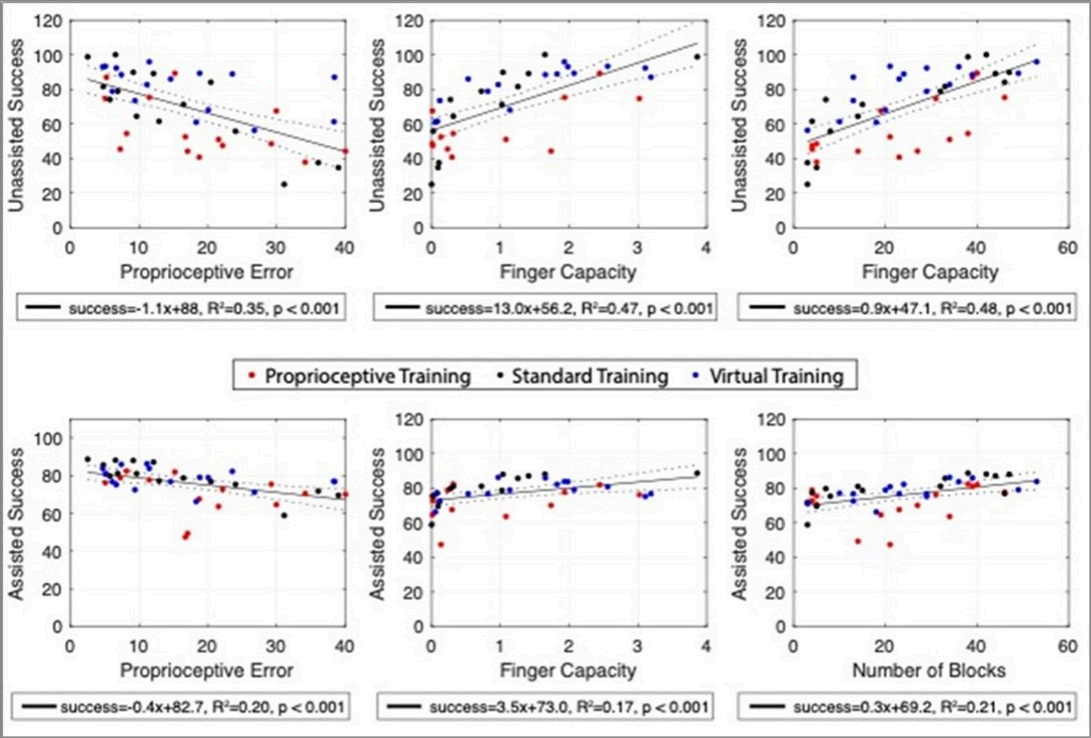
Effect of assistance on gameplay success, plotted as a function of proprioceptive error, finger capacity, and BBT score. Top Row: Considering average unassisted gameplay success across all game modes, both proprioception (Crisscross performance) and motor function (Finger capacity) were significantly related to performance for all training groups. Box and blocks, which depends on both, showed the highest relationship to unassisted performance. Bottom row: With assistance, the relationship to baseline sensorimotor function persisted, but was significantly attenuated, such that individuals with a variety of impairments played all gaming modes with relatively high success (estimated between 70-80%).

With assistance turned off, gameplay success was significantly correlated with finger proprioception and motor ability across all training modalities, diminishing with greater impairment (Fig. 6). With assistance turned on, there was still a significant relationship of success to proprioceptive and motor impairment, however the magnitude of the effect was lower, evidenced by the difference in slope estimations for their respective relationships to unassisted and assisted gameplay (ANCOVA, all p < 0.001; Proprioception to unassisted: −1.10 ± 0.22, and assisted success: −0.38 ± 0.11; Finger Capacity to unassisted: 13.0 ± 2.2, and assisted success: 3.6 ± 1.2;). The BBT score, which presumably depends on both motor and proprioceptive ability, showed the strongest initial relationship to success. However, again, the relationship of success to BBT score relationship was substantially attenuated and success was elevated with assistance (ANCOVA, p < 0.001; BBT to unassisted: 0.96 ± 0.14, and assisted success: 0.28 ± 0.08).

### Self-reported motivation for different training modes

**Figure 7:**
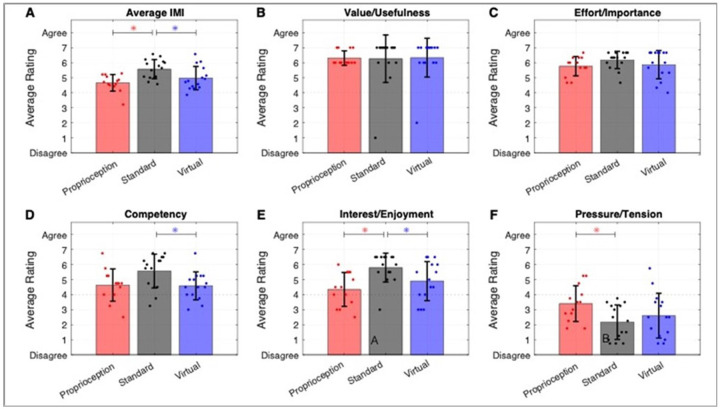
Self-ratings of motivation for training (i.e., IMI scores) across the three training groups. Subplot A shows the overall IMI score, while B-E show the components of the IMI. All groups rated the training as very Valuable/Useful and rated them as activities that were important to them/required effort. Differences between training modes were evidenced in perceived Competency, Interest/Enjoyment, and Pressure/Tension. Asterisks denote significance of pairwise comparisons at p < 0.05

All groups rated their motivation as positive following one week of training (i.e. significantly greater than 4; Average IMI (mean +/− std): Proprioceptive Training: 4.65 +/−0.55, Standard Training: 5.57 +/− 0.64, Virtual Training: 4.97 +/− 0.78). However, participants in the Standard Training group reported significantly greater motivation compared to the Virtual and Proprioceptive Training groups, based on the overall IMI score (Fig. 7A). While the IMI subscales of training Value/Usefulness (Fig. 7B, mean ± std: 6.3 ± 1.2) and Effort/Importance (Fig. 7C, mean ± std: 5.9 ± 0.7) were rated highly and comparably across groups, the other IMI subscales showed significant differences between groups, as detailed below.

We performed further analyses to understand the difference in overall IMI ratings between Standard and Virtual Training. We first note that success was not significantly different between the Standard and Virtual Training groups for any gaming mode (Fig. 3). Thus, the group receiving physical assistance (i.e., Standard Training) reported significantly greater motivation than the group receiving virtual assistance despite matched success levels. When we examined components of the IMI that could account for this difference, we found that the Competency (Fig. 7D) and Interest/Enjoyment components (Fig. 7E) were significantly decreased for Virtual Training compared to Standard Training. One of the sub-questions related to Competency exhibited especially dramatic differences between groups (supplemental Fig. 1): people who engaged in Virtual Training responded to the statement that they “couldn’t do the activity well” with a rating roughly double (3.9 ± 1.8) the physical assistance group (2.1 ± 1.6, t-test p < 0.009) even though the adaptive assistance caused them to have similar game success at both games. Thus, moving better through the action of robotic assistance contributed to an improved self-rating of motivation in physical versus virtual assistance modes, particularly through the domain of Competency.

In the Proprioceptive Training group, differences in overall IMI compared to Standard Training stemmed from decreased ratings in the Interest/Enjoyment (Fig. 7E) and increased Pressure/Tension (Fig. 7F) subscales. While both groups received physical assistance, the Proprioceptive Training group required significantly more assistance to play the games and achieved significantly lower success in RehabHero compared to the Standard Training group. The higher gaming difficulty likely contributed to the higher ratings of Pressure/Tension, while success differences may have contributed to lower ratings of Interest/Enjoyment, supported by a further correlation analysis detailed below. Additionally, for the Interest/Enjoyment sub-scale, we observed that the most significant difference between group ratings occurred on the question “I think that this activity is boring” (proprioceptive: 3.5 ± 2.4 rating; standard: 1.4 ± 1.1 rating, t-test p < 0.002, Supplemental Fig. 1). In the proprioception group, individuals with higher levels of proprioceptive impairment (ThumbSense error > 20%, Crisscross error > 15 deg) tended to rate the game as boring while individuals with low levels of proprioceptive deficit did not (high impairment level (N = 7): 4.85 ± 2.4; low impairment level (N = 8): 2.0 ± 1.1; t-test, p < 0.02). This suggests that individuals with proprioceptive deficit may have somewhat lower engagement with the game due to their inability to accurately perceive the proprioceptive gaming cues. However, two individuals with some of the most severe deficits (ThumbSense error > 35%, Crisscross error > 19 deg) rated their levels of boredom as a 1–2, suggesting this is not a hard-and-fast rule.

**Figure 8:**
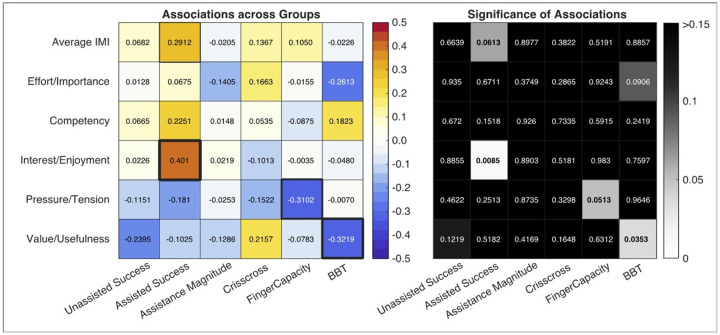
Results of an exploratory correlational analysis of factors (Game Performance, Position sense, Sensorimotor Function) that explain participants intrinsic motivation for training. For this figure, participants in all three training groups are combined to allow a larger sample size for the correlation analysis. Assisted game play success had the strongest relationship to motivational ratings, primarily through the modality of Interest/Enjoyment. Motor function, quantified by finger capacity, was also related to motivation through the modality of Pressure/Tension, with individuals with lower hand capacity feeling greater tension. Sensorimotor function, quantified by Box and Block Test score (BBT), was also related to motivation; BBT was negatively correlated with Value/Usefulness, and weakly with Effort/Importance such that individuals with greater impairment (lower BBT scores) tended to rate the training higher in these categories.

Finally, we performed an exploratory analysis of gameplay features (success, magnitude of assistance) and baseline sensory (Crisscross) and motor function (Finger Capacity), and hand function (BBT) to understand their relationship to the motivation outcomes (Fig. 8). We first analyzed all the groups together. Training success had the strongest relationship with participants’ Interest/Enjoyment of the games (R = 0.4, p < 0.009), with higher success corresponding to greater enjoyment. Across training groups, individuals with lower BBT scores tended to rate the value/usefulness of training higher (R = 0.32, p < 0.035), and individuals with lower finger capacity tended to report higher feelings of pressure/tension during gameplay (R = −0.31, p < 0.051).

Next, we analyzed each training group separately. For the Proprioceptive Training group, Competency was significantly positively associated with assistance gains (R = 0.71, p < 0.007) such that greater assistance was associated with greater feelings of training competency. All other associations were either in line with the group level results (success modulating Interest/Enjoyment, and functional impairment modulating perceived Value/Usefulness of training), or did not reach significance.

## Discussion

We tested the feasibility of two novel robotic therapy modes. The first mode, Virtual Training, uses virtual assistance to decouple gameplay success from movement performance, aiming to allow high levels of success without robotic physical assistance. The second mode, Proprioceptive Training, requires participants to make gameplay decisions based on sensed finger positions, aiming to aid proprioceptive learning. We hypothesized that an adaptive algorithm would be able to adapt levels of success for individuals with a wide range of motor and proprioceptive impairments for both of these training modes, comparable to Standard Robotic Training. Further, we hypothesized that the high success levels endowed by the assistance would lead individuals to rate the training modes as motivating, providing support for their feasibility for long-term training.

The feasibility testing of these modes with 46 people in the chronic phase post-stroke revealed several key findings. The adaptive assistance algorithm effectively adjusted success rates to ~ 80% for all three training modes, even when unassisted game success varied widely across participants. Proprioceptive training required greater levels of assistance, and had lower success without assistance. Participants in the Virtual Training group reported significantly lower motivation compared to those in the physical training group, despite achieving comparable success levels. This lower motivation rating was primarily linked to lower perceived competence, while across all training groups, success related to perceived Interest/Enjoyment. We discuss now these findings, as well as directions for future research.

### Virtual Training modulated gameplay success but did not fully support motivation

For Virtual Training, in which only virtual computer representations of movement were altered to achieve success, the adaptive controller was able to reliably achieve the target game play success of 80%. This was true for individuals with a wide range of motor impairment, as evidenced by the widely varying success rate in unassisted gameplay, during which individuals with lower BBT scores, for example, had lower gameplay success. With virtual assistance, the dependence of game success on BBT score was significantly reduced, such that even individuals with low-to-moderate hand function (BBT < 15) were able to achieve a success rate of 70% or greater. Moreover, for individuals in this group with high levels of success (> 90%), the adaptive algorithm was able to increase the challenge level, such that the training did not become too easy, potentially limiting its perceived Value/Usefullness as observed in our randomized control trial of sensorized home-based training [31]. Thus, a simple adaptive strategy that adjusts virtual computer representations of movement can effectively control game success. Note that active actuation is not necessary in this Virtual Training mode, such that it can be implemented using much simpler sensor-based devices or camera vision technology.

While the Virtual Training group rated their motivation positively, it was significantly lower than the Physical Training group, even though their success levels were comparable at the group level. Their lower overall motivation rating was attributable to lower rankings in the sub-category of perceived competence. As the only difference between groups was the lack of physical assistance to help participants to physically perform the cued movements, we interpret these results to indicate that physical assistance increases feelings of competency by helping to realign volitional movement intent with actual physical movement. This provides novel evidence that the physical assistance made possible by active actuation contributes to subjective ratings of motivation for training, mainly though movement assistance increasing participant feelings of competency.

### Proprioceptive Training was feasible even for individuals with proprioceptive impairments

It was not apparent to us at the onset of this work that asking people to play robotic therapy games that incorporate proprioceptive decision making would be feasible for people who have proprioceptive impairment. This is somewhat analogous to asking a person with visual impairment to play a visually guided game. However, the adaptive assistance strategy we implemented was capable of achieving the target gameplay success of ~ 80% for the Proprioceptive Training group, reducing the dependence of gameplay success on sensory as well as motor impairment.

As a result, individuals rated Proprioceptive Training nearly as motivating as those experiencing Standard Training, although there was a small but significant decrease in IMI score. We believe this is because the Proprioceptive Training mode was comparatively more challenging than Standard Training, as evidenced by the significantly higher physical assistance needed to play both RehabHero and FingerPong, and the slightly lower success achieved in RehabHero. As roughly half of the participants in this group experienced substantial proprioceptive impairment as quantified by the robotic assessments, it is likely that the requirement to make gameplay decisions based on proprioceptive input caused increased gaming difficulty and contributed to feelings of boredom for some individuals who could not reliably sense these cues. Exploratory linear regression analysis in the Proprioceptive Training group indicated that physical assistance significantly improved feelings of competency, suggesting that physical assistance successfully mediated feelings of incompetence/challenge during unassisted gameplay. These results are in line with results observed in the Virtual Training group, indicating the physical assistance helps improve feelings of Competency by helping participants perform the cued movements during training. Finally, while this game required proprioceptive decision making, we saw that with assistance, even severely impaired individuals were able to play the game at high success rates, making it a novel sensory-motor training methodology applicable to a broad range of individuals post-stroke.

### Broader observations and future directions

We previously found that gameplay success alters self-ratings of motivation for robotic training [28]. Here, we found that gameplay success across all groups was a significant predictor of the Interest/Enjoyment component of the IMI, even when movement was being assisted. This provides new insight into how gameplay success affects overall self-ratings of motivation: success makes gameplay more enjoyable and interesting. In contrast, as described above, physical assistance appears to improve motivation by improving feelings of competency.

We expected proprioceptive ability to affect gameplay success for Proprioceptive Training, but here we also found that it predicted both unassisted and assisted success in both visually guided modes that provided virtual or physical assistance. One possibility is that proprioceptive ability plays a fundamental role in movement control and is thus predictive of performance in a broad diversity of forms of movement training. Beyond motor control, proprioception is linked to one’s ability to learn and benefit from movement training [28]. We found previously that proprioceptive ability was the most important predictor of the therapeutic response to robotic hand training [29]. Improving our understanding of the roles of proprioception in movement rehabilitation is an important direction for future research.

Finally, at the time of submission of this paper, we had not yet completed the associated randomized clinical trial in which the participants in this paper completed three weeks of robotic hand training with one of the training strategies – i.e., either Standard, Virtual, or Proprioceptive Training. We are currently analyzing the therapeutic effects of these strategies as a function of the baseline sensory and motor impairments of the participants and will report them in a companion paper.

## Conclusion

Here, we demonstrated that novel Virtual Assistance and Proprioceptive Training modes are feasible for post-stroke individuals with a wide range of chronic motor and proprioceptive impairment. The simple assistance algorithm controlled success effectively such that participants in all training modes were able to achieve high gameplay success and motivation for training. Success across all gaming modalities was related to participants’ motor function, but also proprioceptive function. While proprioceptive gaming success had a relatively higher association to proprioceptive ability, proprioceptive ability predicted unassisted and assisted success in both visually guided modes, consistent with the idea that proprioceptive ability plays a fundamental role in these types of movements and games. The results also highlight the importance of success modulation in sustaining motivation, with gameplay success being the strongest predictor of participants' training enjoyment among six assessed features of gameplay and sensorimotor function. Virtual Assistance Training controlled success without physical assistance, making its application feasible with simpler sensor-based systems. However, results showed that physical assistance contributes to raising training motivation by increasing participants’ perceived competency, giving physical assistance strategies a slight advantage over virtual assistance in terms of participants' motivation. Finally, Proprioceptive Training, though more challenging, remained motivating with high levels of assistance that made it viable even for those with proprioceptive impairments.

## Figures and Tables

**Table 1:** Participant characteristics. Numbers are mean ± std

Training Group	All (N = 46)	Proprioception (N = 15)	Standard (N = 15)	Virtual (N = 16)
Age	57 ± 15	61.9 ± 13.6	54.3 ± 16.9	55 ± 14.2
Days post stroke	1688 ± 1276	1614 ± 1264	2051 ± 1271	1417 ± 1290
Gender [M/F]	[34/12]	[10/5]	[11/4]	[14/2]
Side of Paresis [R/L]	[20/26]	[7/8]	[6/9]	[7/9]
Type of Stroke [Hemorrhagic/Ischemic]	[19/27]	[4/11]	[7/8]	[8/8]

**Table 2:** Participants sensorimotor function assessed clinically and robotically. Numbers are mean ± std

Training Mode				
	All (N = 46)	Proprioception (N = 15)	Standard (N = 15)	Virtual (N = 16)
UE Fugl-Meyer	46.0 ± 11.1	45.0 ± 9.8	41.7 ± 13.1	50.9 ± 8.7
Box and Block [blocks]	24.0 ± 15.2	23.3 ± 14.6	22.7 ± 17.8	25.9 ± 13.8
Finger Capacity [n.u.]	1.1 ± 1.1	0.94 ± 1.08	0.85 ± 1.04	1.47 ± 1.04
Crisscross [deg]	12.5 ± 5.9	13.2 ± 5.4	11.6 ± 6.4	12.5 ± 6.2
Move and Match [deg]	8.6 ± 3.3	9.2 ± 3.3	8.2 ± 3.4	8.4 ± 3.4
ThumbSense [% accurate]	78.5 ± 17.6	75 ± 17.9	80 ± 18.2	80 ± 17.5
Movements Attempted	3013 ± 307	2915 ± 452	2987 ± 265	3127 ± 58
